# Supplementary material for: SMAP is a pipeline for sample matching in proteogenomics
Source: Nat Commun. 2022 Feb 8;13:744. doi: 10.1038/s41467-022-28411-8 (PMC8825821; doi:10.1038/s41467-022-28411-8)
Supplement: Supplementary file 1 — Supplementary Information [file 41467_2022_28411_MOESM1_ESM.pdf]

Supplementary information for

SMAP is a pipeline for sample matching in proteogenomics

Ling Li, Mingming Niu, Alyssa Erickson, Jie Luo, Kincaid Rowbotham, Kai Guo, He Huang, Yuxin Li, Yi Jiang, Junguk Hur, Chunyu Liu, Junmin Peng, Xusheng Wang

Supplementary Figures

Supplementary Fig. 1. Workflow of the SMAP pipeline.

Supplementary Fig. 2. Standardizing intensities and inferring sample genotypes.

Supplementary Fig. 3. Strategy of genotype assignment.

Supplementary Fig. 4. Flowchart showing the strategy of sample shuffling.

Supplementary Fig. 5. Evaluation of scoring strategy.

Supplementary Fig. 6. Performance evaluation of SMAP.

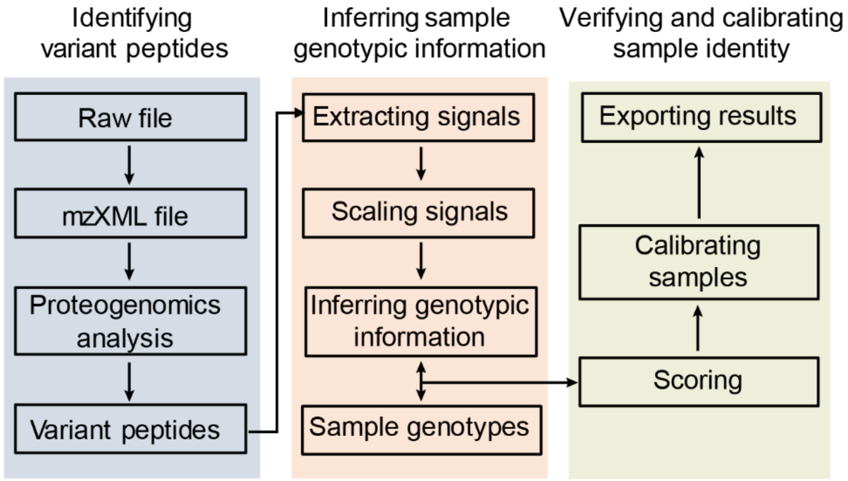

Supplementary Fig. 1. Workflow of the SMAP pipeline.

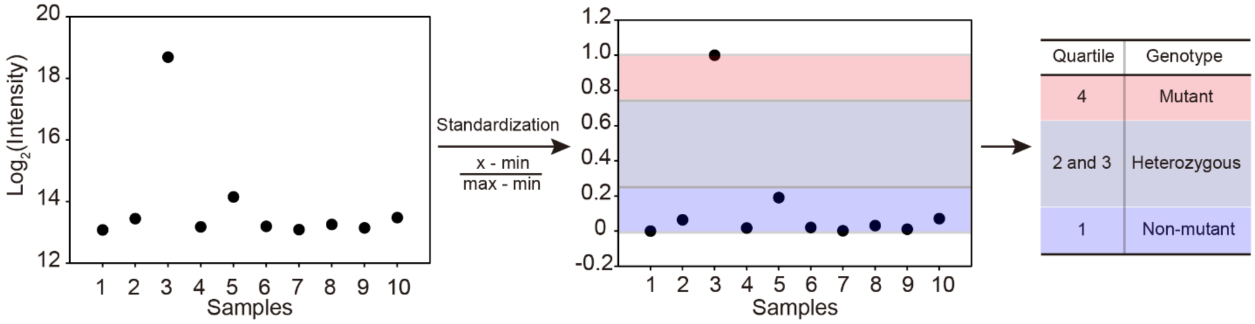

Supplementary Fig. 2. Standardizing intensities and Inferring sample genotypes. The expression levels of a variant peptide were scaled to the range of 0 to 1. The scaled expression levels are divided into three quartiles: homozygous non-mutant genotype, heterozygous genotype, and homozygous mutant genotype.

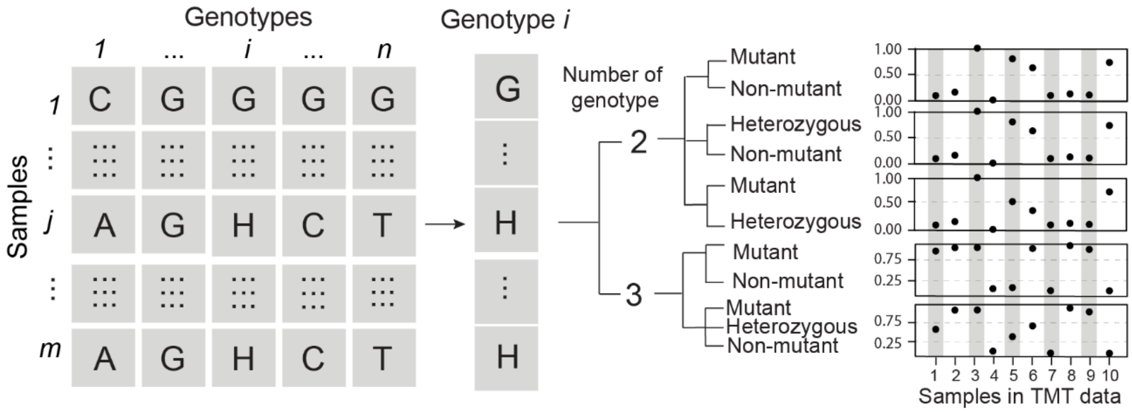

Supplementary Fig. 3. Strategy of genotype assignment. SMAP determines the number of genotypes as prior knowledge to assign inferred genotypes. With the genotype number, SMAP divides the scaled intensities into either quartile (homozygous non-mutant, heterozygous, and homozygous mutant) or dichotomy (homozygous non-mutant and mutant).

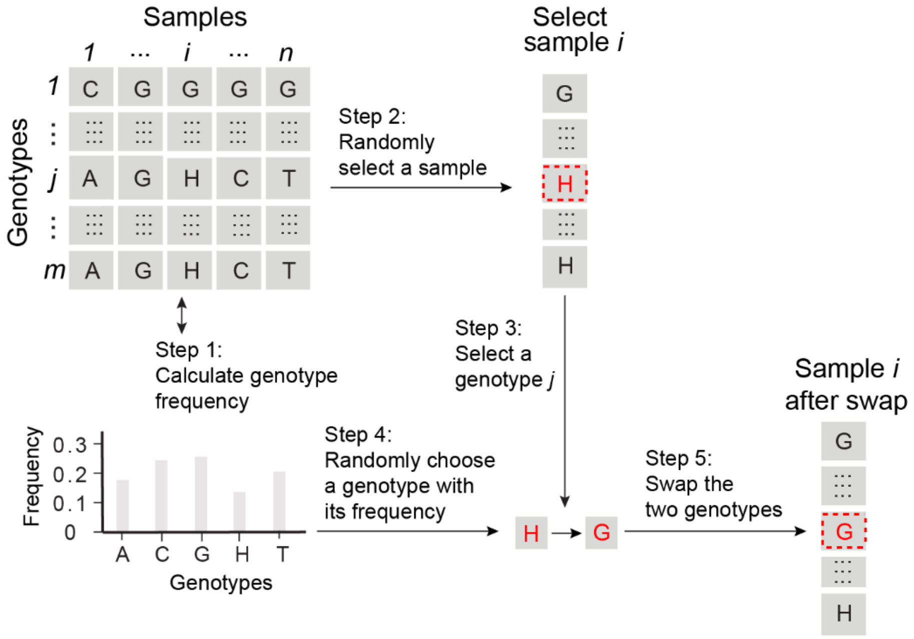

Supplementary Fig. 4. **Flowchart showing the strategy of sample shuffling.** A simulated dataset is generated in six steps: (1) estimating the frequency of each genotype from all SNPs across all samples; (2) randomly selecting a sample i; (3) randomly select a genotype j in sample i; (4) choosing another genotype with its frequency estimated in step 1; (5) swapping the genotype j in sample i with a chosen genotype in step 4; and (6) repeating steps 1-5 to generate a simulated dataset with a certain percentage of the error rate.

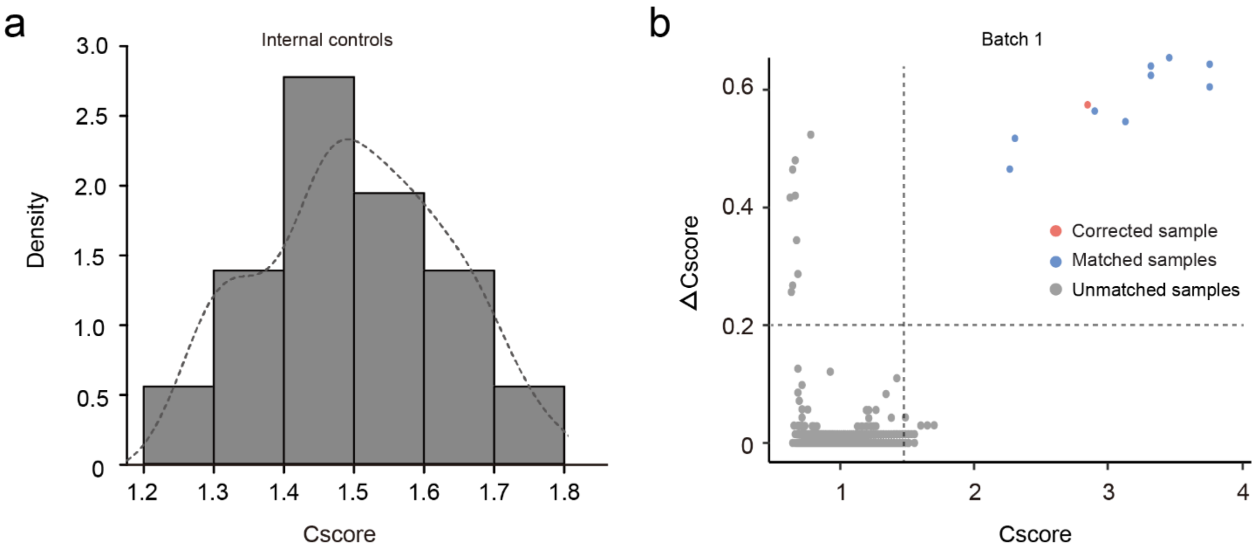

Supplementary Fig. 5. Evaluation of scoring strategy. (a) Distribution of Cscore in internal controls. (b) Distribution of Cscore and ΔCscore when applying SMAP to one batch TMT proteomic dataset.

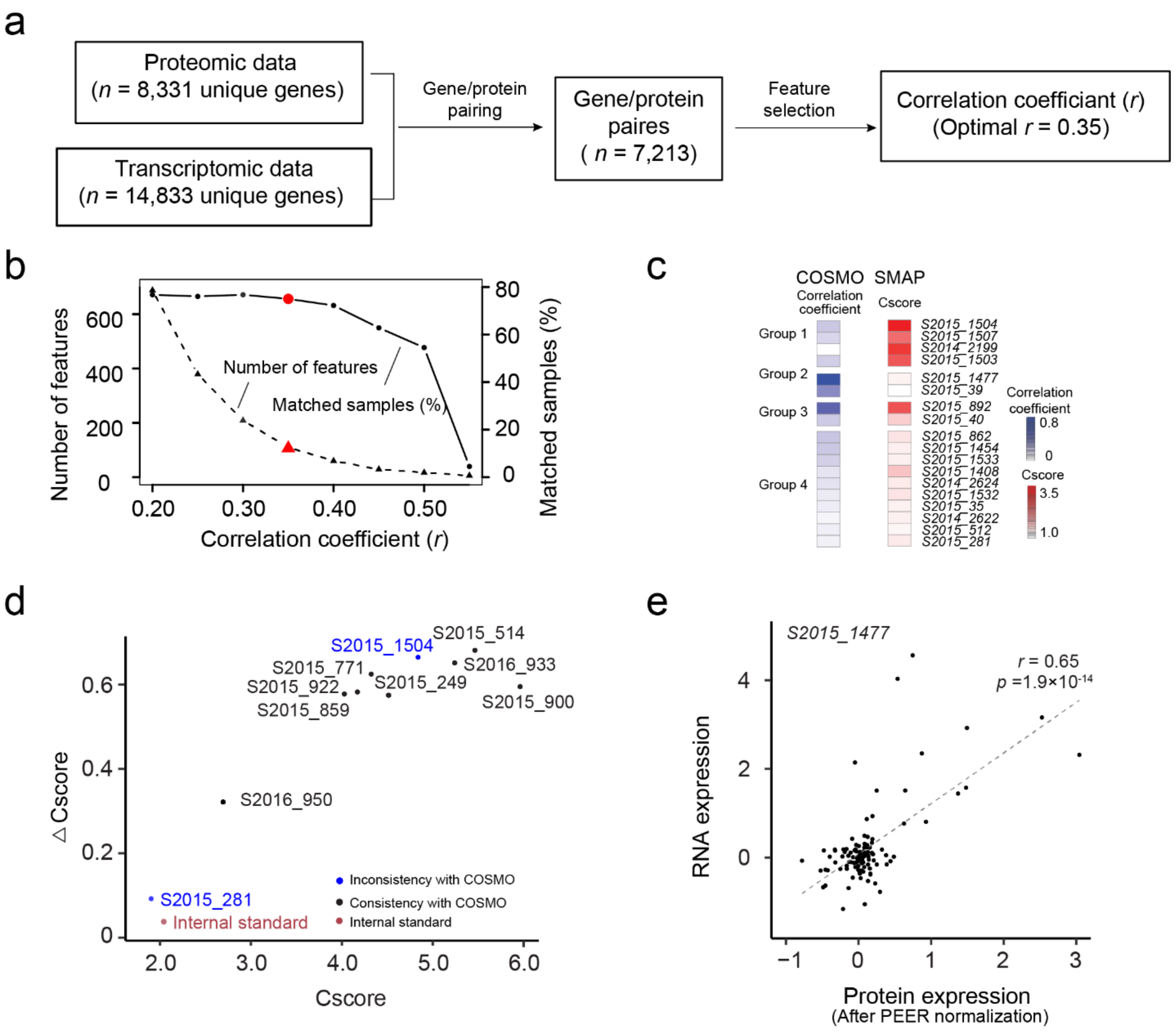

Supplementary Fig. 6. Performance evaluation of SMAP. (a) Flowchart showing the selection of optimal correlation coefficient. (b) Evaluation of correlation coefficient in COSMO based on the number of selected features and the percentage of matched samples. (c) Heatmap showing inconsistent corrections between COSMO and SMAP. Group 1: Four samples matched to the original identity were "mis-assigned" to the other identity by COSMO, whereas SMAP made no such correction; Group 2: Two samples were "mis-assigned" by SMAP, but COSMO did not make a correction; Group 3: Two samples were adjusted into different sample identities by both SMAP and COSMO with high scores; Group 4: Corrections made by both SMAP and COSMO with low scores. (d) Scatter plot showing the distribution of Cscore and ΔCscore for 11 samples in one TMT batch. (e) Scatter plot showing the correlation between expression levels of protein and RNA for the sample *S2015_1477*. Correlation of mRNA-protein pairs was calculated using Pearson's correlation and two-tailed *p* value.
