# Supplementary material for: SMAP is a pipeline for sample matching in proteogenomics
Source: Nat Commun. 2022 Feb 8;13:744. doi: 10.1038/s41467-022-28411-8 (PMC8825821; doi:10.1038/s41467-022-28411-8)
Supplement: Supplementary file 3 — Description of Additional Supplementary Files [file 41467_2022_28411_MOESM3_ESM.pdf]

Description of Additional Supplementary Files

File Name: Supplementary Data 1
Description: Variant peptides and genotypes identified in the PsychENCODE BrainGVEX proteomics project

File Name: Supplementary Data 2
Description: Summary of the variant peptides
